# Neurotrophic Control of Puberty: From Molecular Signaling to Disorders of Pubertal Timing

**DOI:** 10.3390/cimb48010003

**Published:** 2025-12-19

**Authors:** Roberto Paparella, Norma Iafrate, Roberta Lucibello, Arianna Bei, Irene Bernabei, Cinzia Fiorentini, Lavinia Marchetti, Francesca Pastore, Vittorio Maglione, Marcello Niceta, Marco Fiore, Sabrina Venditti, Ida Pucarelli, Luigi Tarani

**Affiliations:** 1 Department of Maternal Infantile and Urological Sciences, Sapienza University of Rome, 00185 Rome, Italy; 2 Molecular Genetics and Functional Genomics, Ospedale Pediatrico Bambino Gesù, IRCCS (Istituto di Ricovero e Cura a Carattere Scientifico), 00165 Rome, Italy; 3 Institute of Biochemistry and Cell Biology (IBBC-CNR), c/o Department of Sensory Organs, Sapienza University of Rome, 00185 Rome, Italy; 4 Department of Biology and Biotechnologies “Charles Darwin”, Sapienza University of Rome, 00185 Rome, Italy

**Keywords:** neurotrophins, BDNF, NGF, precocious puberty, delayed puberty, hypogonadotropic hypogonadism

## Abstract

The onset of puberty is a critical developmental milestone regulated by complex neuroendocrine networks that integrate genetic, metabolic, and environmental cues. Among the molecular systems coordinating this transition, neurotrophins—including brain-derived neurotrophic factor (BDNF), nerve growth factor, neurotrophin-3, and neurotrophin-4/5—have emerged as important modulators of hypothalamic maturation and the activation of gonadotropin-releasing hormone (GnRH) neurons. Beyond their established roles in neuronal survival and differentiation, neurotrophins contribute to hypothalamic circuit plasticity, influence GnRH neuronal activity, and participate in the integration of metabolic and environmental signals relevant to reproductive maturation. Experimental studies, primarily based on animal and cellular models, demonstrate that BDNF and its receptor play a role in normal pubertal onset, whereas disruptions in neurotrophin signaling have been implicated in central precocious puberty, delayed puberty, and hypogonadotropic hypogonadism. In humans, available evidence is more limited and derives mainly from genetic studies, circulating neurotrophin measurements, and clinical observations. This review provides an integrative synthesis of current experimental and clinical data on neurotrophin-mediated regulation of pubertal timing, highlighting both physiological mechanisms and pathological conditions. While neurotrophins represent promising modulators at the intersection of neurodevelopment, metabolism, and reproduction, further longitudinal and translational human studies are required to define their diagnostic and therapeutic potential in pediatric endocrinology.

## 1. Introduction

Puberty is a fundamental developmental transition that marks the shift from a prepubertal state to full reproductive maturity. This process relies on the activation of the hypothalamic–pituitary–gonadal (HPG) axis, which is tightly regulated by a coordinated interplay of neuroendocrine and metabolic signals. Its timing depends on the balance between inhibitory mechanisms that maintain childhood quiescence and stimulatory cues that later reactivate gonadotropin-releasing hormone (GnRH) secretion [1,2]. Traditionally, research has focused on key regulators such as kisspeptin, GnRH itself, and leptin—molecules that serve as central integrators of reproductive and metabolic status. Kisspeptin provides the strongest known stimulus for GnRH pulsatility, while leptin conveys information on energy sufficiency, ensuring adequate metabolic resources to support reproductive maturation [3,4,5].

In recent years, this classical neuroendocrine framework has been expanded by the recognition of an essential role for neurotrophic factors, particularly brain-derived neurotrophic factor (BDNF) and related neurotrophins. Initially characterized for their involvement in neuronal survival, differentiation, and synaptic plasticity, neurotrophins are now understood to influence the reproductive axis at multiple levels [6]. BDNF, for example, modulates GnRH neuronal activity and gene expression through specific intracellular signaling pathways, acting as a functional bridge between neural architecture, environmental inputs, and endocrine signals [7]. This adds an important layer of complexity to the regulation of pubertal timing, highlighting how neuronal plasticity and neurotrophic support shape the transition toward reproductive competence. This integrative perspective reframes puberty as a process in which neurotrophic modulation intersects with classical neuroendocrine and metabolic pathways to orchestrate HPG axis activation. Disruptions in these interactions can consequently lead to disorders of pubertal timing, including central precocious puberty, delayed puberty, and hypogonadotropic hypogonadism [8,9].

Despite extensive research on the HPG axis and its canonical regulators, no prior integrative review has specifically focused on neurotrophins as modulators of pubertal timing across both physiological development and clinical disorders. Moreover, recent years have witnessed a rapid expansion of experimental and translational data linking neurotrophic pathways to GnRH neuron function, hypothalamic plasticity, and distinct pubertal phenotypes. These advances create a timely opportunity to reframe neurotrophins not merely as developmental factors, but as dynamic integrators of neurodevelopmental, metabolic, and reproductive signals relevant to disorders of pubertal timing. This review provides a conceptual and translational framework for understanding how neurotrophic signaling shapes pubertal timing. Rather than describing neurotrophins as isolated developmental factors, we focus on their dynamic role in orchestrating hypothalamic plasticity, GnRH neuron activation, and network-level integration during reproductive maturation. By jointly addressing physiological puberty and its pathological extremes—including central precocious puberty, delayed puberty, congenital hypogonadotropic hypogonadism, and functional hypothalamic amenorrhea—this review offers a unified perspective that links molecular neurotrophic pathways to clinical phenotypes of pubertal dysregulation.

This article is a narrative and integrative review aimed at synthesizing current evidence on the role of neurotrophic signaling in pubertal regulation. The literature was identified through targeted searches of PubMed, Scopus, and Web of Science using combinations of keywords related to neurotrophins, GnRH neurons, puberty, hypothalamic plasticity, and pubertal disorders. Priority was given to peer-reviewed experimental, translational, and clinical studies published in English. Rather than following a systematic review protocol, the selected literature was critically evaluated and conceptually integrated to develop a unifying framework linking molecular neurotrophic pathways, hypothalamic network remodeling, and clinical phenotypes of altered pubertal timing.

## 2. Molecular Biology and Physiology of Neurotrophins

### 2.1. Overview of the Neurotrophin Family

Neurotrophins constitute a highly conserved family of growth factors essential for the development, maintenance, and plasticity of the nervous system. The four principal members—nerve growth factor (NGF), BDNF, neurotrophin-3 (NT-3), and neurotrophin-4/5 (NT-4/5)—are synthesized as precursor proteins (proneurotrophins) within neuronal and glial cells and subsequently cleaved into their biologically active mature forms [10,11,12,13,14,15,16]. Once released, neurotrophins exert their effects through selective interactions with high-affinity tropomyosin receptor kinases (TrkA, TrkB, TrkC) and the low-affinity p75 neurotrophin receptor (p75NTR). NGF preferentially binds TrkA, BDNF and NT-4/5 bind TrkB, and NT-3 primarily activates TrkC, although some degree of cross-reactivity exists [14,17]. The p75NTR receptor, is a member of the Tumor Necrosis Factor receptor superfamily, and is capable of binding all neurotrophins, functions as a modulatory receptor that can bias signaling toward survival or apoptosis depending on receptor context, ligand form, and intracellular milieu [15,18,19,20]. This functional diversification establishes neurotrophins as key regulators of neuronal development and circuit refinement.

### 2.2. Neurotrophin Signaling Pathways

Activation of Trk receptors by their respective ligands initiates a limited set of canonical intracellular signaling pathways that collectively regulate neuronal survival, differentiation, and synaptic plasticity [11,21]. The three principal cascades downstream of Trk receptors are the mitogen-activated protein kinase/extracellular signal-regulated kinase (MAPK/ERK) pathway [22], the phosphoinositide 3-kinase/protein kinase B (PI3K/Akt) pathway [23], and the phospholipase C gamma (PLCγ) pathway [24]. Together, these pathways coordinate transcriptional programs, cytoskeletal dynamics, and activity-dependent synaptic modifications essential for nervous system development and function.

In contrast, signaling through the low-affinity p75 neurotrophin receptor is highly context dependent. When activated by proneurotrophins or in the absence of Trk co-signaling, p75NTR can engage adaptor proteins that promote apoptotic signaling, growth cone retraction, and inhibition of neurite extension [25,26,27,28]. The relative balance between Trk-mediated and p75NTR-mediated signaling therefore allows neurotrophins to finely tune neuronal architecture and circuit refinement during development and across the lifespan [15].

### 2.3. Expression Patterns During Development

Neurotrophin and receptor expressions are dynamically regulated during central nervous system development, particularly in hypothalamic and limbic structures involved in endocrine control. Evidence from murine models demonstrate that, in the prepubertal hypothalamus, NT-3 is most highly expressed during early neuronal proliferation and migration, supporting precursor differentiation and initial circuit formation. As regions mature, NT-3 expression declines, while BDNF expression increases, coinciding with synaptic maturation and the establishment of functional neuronal networks. NGF shows variable expression, contributing to the survival and differentiation of select neuronal populations, but is less consistently linked to GnRH circuit maturation than NT-3 and BDNF [29].

BDNF and its receptor TrkB show marked upregulation in the arcuate nucleus and preoptic area during the transition from childhood quiescence to the onset of pulsatile GnRH secretion. This neurotrophic surge coincides with enhanced synaptic plasticity and increased sensitivity to metabolic and environmental signals, providing a permissive neurobiological substrate for the initiation of puberty [7,16,30,31,32].

In parallel, neurotrophin expressions within the limbic system undergo reorganization during adolescence, influencing networks related to emotional regulation, reward processing, and social behavior—domains that undergo marked transformation during this developmental window [33,34,35]. These temporally coordinated neurotrophin dynamics underscore their fundamental role not only in neurodevelopment but also in reproductive maturation and adolescent behavioral adaptation.

## 3. Neurotrophins and the Onset of Puberty: Molecular Mechanisms

### 3.1. BDNF–TrkB Signaling and GnRH Neuron Activation

BDNF and its high-affinity receptor TrkB play an essential role in the activation and maturation of GnRH neurons, which represent the central drivers of pubertal initiation. BDNF acts via TrkB to stimulate intracellular signaling cascades, most notably the PI3K/Akt pathway, which promotes GnRH gene expression and neuronal activity. Experimental evidence demonstrates that BDNF directly increases GnRH expression in hypothalamic neurons, and upregulation of BDNF/TrkB signaling accelerates puberty onset by enhancing GnRH release and activating the HPG axis [31]. Conversely, suppression of BDNF or TrkB signaling delays pubertal onset and reduces GnRH output.

Most mechanistic insights into BDNF–TrkB signaling in GnRH neurons derive from preclinical studies, primarily in rodent models. GnRH neurons express TrkB receptors, enabling them to directly respond to BDNF-mediated cues. BDNF binding to TrkB activates canonical TrkB-dependent signaling pathways, supporting neuronal survival, neurite outgrowth, and functional maturation of GnRH neurons [36,37]. In embryonic GnRH neurons, BDNF induces neurite extension and cyclic adenosine monophosphate (cAMP) response element-binding protein (CREB) phosphorylation, facilitating the development of the GnRH secretory system [36]. BDNF also acts on glutamatergic circuits upstream of GnRH neurons, enhancing excitatory synaptic input and thereby increasing GnRH neuronal firing and pulsatility [30,38]. At glutamatergic synapses, BDNF-TrkB signaling regulates presynaptic glutamate release and postsynaptic receptor phosphorylation, which strengthens synaptic transmission and plasticity [38]. This activity-dependent modulation of glutamatergic input is critical for the dynamic regulation of GnRH neuron activity and the initiation of puberty.

### 3.2. Neurotrophins and Kisspeptin System Crosstalk

Accumulating evidence highlights a functional interplay between neurotrophins and the kisspeptin system, one of the most potent activators of GnRH secretion. Kisspeptin-producing kisspeptin/neurokinin B/dynorphin (KNDy) neurons integrate metabolic, hormonal and neurotrophic signals to regulate GnRH pulsatility and neuronal network maturation through several mechanisms. These neurons, located in the arcuate nucleus, co-express kisspeptin, neurokinin B, and dynorphin, and act as the central “pulse generator” for GnRH secretion [39,40,41]. KNDy neurons receive direct inputs from metabolic hormones such as leptin and insulin, which signal energy sufficiency and modulate kisspeptin expression and neuronal excitability [42,43,44]. The functional characterization of KNDy neuron interactions with neurotrophins is largely based on experimental evidence from rodent models, while direct human data remain limited.

Sex steroids provide feedback regulation, with estradiol exerting differential effects on kisspeptin synthesis in distinct hypothalamic populations [42,44]. Neurotrophic factors like BDNF further support the structural and functional maturation of KNDy-GnRH circuits, enhancing synaptic connectivity and responsiveness to metabolic and hormonal cues [45]. Within this framework, BDNF–TrkB signaling intersects with KNDy neuron function by promoting synaptic stability, dendritic remodeling, and excitatory/inhibitory balance. This crosstalk ensures efficient neuroendocrine transmission to GnRH neurons and contributes to the robust reactivation of GnRH pulsatility that characterizes the onset of puberty.

### 3.3. Synaptic Remodeling and Neurogenesis

Neurotrophins play a central role in the neurobiological maturation that enables the timely onset of puberty by driving synaptic remodeling, neurogenesis, and the reorganization of excitatory and inhibitory networks within hypothalamic circuits. Evidence supporting neurotrophin-driven synaptic remodeling and neurogenesis during pubertal maturation is predominantly derived from animal models. During the prepubertal-to-pubertal transition, neurotrophins such as BDNF, NGF, and NT-3 become upregulated in key hypothalamic nuclei, where they act through receptors including TrkB to promote neuronal survival, differentiation, dendritic branching, and synaptic plasticity [7,46]. In both GnRH neurons and upstream KNDy neurons, BDNF enhances excitatory glutamatergic inputs while reducing inhibitory gamma aminobutyric acid (GABA)-ergic and dynorphinergic tone, shifting the network balance toward excitation—a prerequisite for initiating pulsatile GnRH secretion [47,48,49].

**Figure 1:**
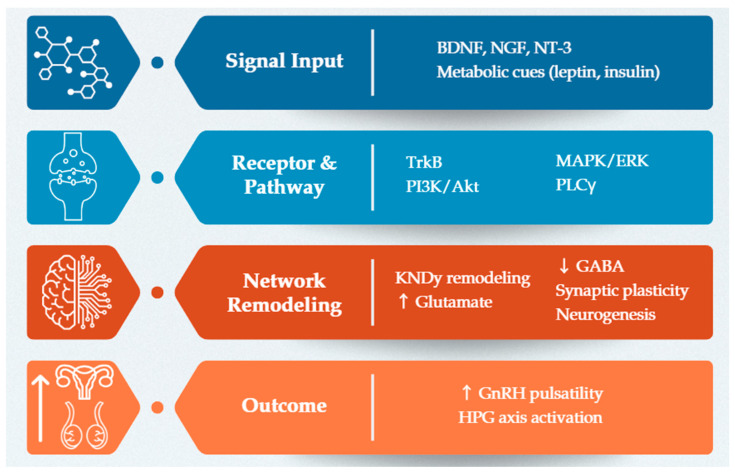
Multilayer model illustrates how neurotrophic signaling regulates pubertal initiation. The diagram shows four hierarchical layers that progressively link neurotrophin input to reproductive output. Layer 1 (Signal Input): neurotrophins and metabolic cues provide upstream regulatory signals. Layer 2 (Receptors and Pathways): activation of TrkB receptors engages intracellular signaling cascades. Layer 3 (Network Remodeling): neurotrophins promote KNDy neuron remodeling, increase glutamatergic excitation, reduce GABAergic inhibition, enhance synaptic plasticity, and support neurogenesis within hypothalamic circuits. Layer 4 (Outcomes): these integrated mechanisms ultimately increase GnRH pulsatility and lead to activation of the hypothalamic–pituitary–gonadal (HPG) axis, facilitating pubertal onset. ↑ indicates elevation. ↓ indicates reduction.

Neurotrophins also support neurogenesis and the incorporation of new neurons into reproductive regulatory circuits, while glial-derived trophic factors facilitate glia–neuron communication essential for synaptic turnover and the release of bioactive molecules that sustain GnRH neuron function. Through activation of canonical neurotrophin-dependent signaling pathways, neurotrophins establish the structural and functional architecture required for coordinated GnRH pulsatility [36,50]. This neurotrophin-dependent plasticity allows hypothalamic networks to dynamically integrate metabolic, hormonal, and environmental cues, enabling the transition from juvenile quiescence to a mature reproductive axis and ensuring that the HPG system remains adaptable to internal physiological states and external challenges [46,51,52] (Figure 1).

## 4. Metabolic Integration: Linking Energy Balance to Reproductive Maturation

### 4.1. BDNF as a Mediator Between Metabolism and Puberty

BDNF serves as a crucial integrator of metabolic cues and reproductive maturation. While epidemiological studies in humans support a permissive role of adiposity and leptin in pubertal timing, much of the mechanistic evidence linking leptin–BDNF signaling to GnRH activation derives from animal and cellular models. BDNF interacts with leptin signaling to influence the activation of hypothalamic circuits governing puberty onset. Leptin, an adipocyte-derived hormone, signals energy sufficiency to the hypothalamus and is permissive for pubertal progression [3,53]. Leptin supplementation increases hypothalamic BDNF expression and related transcriptional regulators, particularly in females, and imprints hypothalamic gene expression patterns that favor energy balance and reproductive maturation [54]. Leptin’s effects on puberty are mediated in part by its ability to upregulate BDNF, which then acts via TrkB receptors to enhance GnRH neuron activation and facilitate the onset of puberty [31,54]. Experimental models show that leptin and BDNF act synergistically to promote the maturation of excitatory networks upstream of GnRH neurons, including kisspeptin and glutamatergic circuits [31,53,54].

Reduced BDNF expression or altered metabolic states (such as energy deficiency or leptin deficiency) impair GnRH release and delay pubertal timing. In animal models, knockdown of BDNF or its upstream regulator fat mass and obesity-associated (FTO) protein in the arcuate nucleus suppresses BDNF/PI3K/Akt signaling, leading to reduced GnRH expression and delayed puberty [31]. Conversely, BDNF overexpression or increased leptin signaling accelerates GnRH release and advances pubertal onset [31,54]. Energy deficit, malnutrition, or leptin deficiency suppresses BDNF and kisspeptin expression, resulting in delayed or absent pubertal progression [3,53]. These mechanistic insights are largely based on experimental models, whereas corresponding human data remain limited and indirect. However, these findings position BDNF as a potential molecular intermediary linking energy balance to the timing of reproductive maturation.

### 4.2. Peripheral Neurotrophins and Gonadal Maturation

Although neurotrophins are best known for their central actions, they also exert important effects in peripheral reproductive tissues, including the ovaries and testes. In the ovary, neurotrophins and their receptors are expressed in granulosa cells, theca cells, and oocytes. BDNF, NGF, NT-3, and NT-4 contribute to follicular development by supporting primordial follicle assembly, follicle recruitment, and growth, as well as promoting granulosa cell proliferation and survival [55,56,57,58]. BDNF and NGF are critical for oocyte maturation, acting via TrkB and TrkA receptors on oocytes and granulosa cells to enhance developmental competence and meiotic progression [59,60,61].

Beyond follicle development, neurotrophins modulate ovarian steroidogenesis. Experimental studies indicate that BDNF stimulates estradiol and progesterone production in granulosa cells by enhancing follicle-stimulating hormone–dependent steroidogenic activity and upregulating key enzymes involved in sex steroid biosynthesis, including aromatase and steroidogenic acute regulatory protein (STAR) [62,63,64]. NGF and NT-3 similarly promote steroidogenic activity and follicular cell proliferation [56,57]. In addition, BDNF acts as an autocrine/paracrine regulator, enhancing granulosa cell proliferation, reducing apoptosis, and antagonizing kisspeptin-mediated suppression of steroidogenesis [64].

In the testes, neurotrophins and their receptors are expressed in Sertoli cells, Leydig cells, and germ cells. NGF and BDNF support Sertoli cell function and germ cell survival, contributing to the regulation of spermatogenesis and testicular development [65,66]. Both neurotrophins also promote Leydig cell proliferation and testosterone production by upregulating key components of the steroidogenic machinery, including STAR, 3β-hydroxysteroid dehydrogenase type 1, and cholesterol side-chain cleavage enzyme [67,68]. NGF exposure increases steroid production in Leydig cells, indicating a direct role in androgen biosynthesis [68].

These peripheral actions complement central neurotrophin signaling, forming a bidirectional communication axis between the hypothalamus and the gonads. The coordinated action of neurotrophins in both compartments ensures the proper progression of pubertal maturation and the establishment of fully functional reproductive capacity.

### 4.3. Adiposity, Neurotrophins, and Timing of Puberty

Body adiposity is a well-recognized determinant of pubertal timing, and neurotrophins appear to mediate part of this relationship. Increased fat mass in early childhood is associated with elevated BDNF expression in the hypothalamus, which promotes GnRH neuron activation and accelerates pubertal onset, including earlier menarche and faster pubertal progression in girls. This is supported by evidence showing that girls with central precocious puberty have higher serum BDNF levels, and experimental models demonstrate that hypothalamic BDNF upregulation (via FTO-mediated N6-methyladenosine (m^6^A) demethylation) stimulates GnRH expression through the PI3K/Akt pathway, leading to earlier puberty onset [31].

Conversely, insufficient adiposity or metabolic dysregulation is associated with reduced BDNF levels, impaired hypothalamic function, and delayed puberty. Chronic energy deficiency, malnutrition, or conditions such as anorexia suppress BDNF and leptin signaling, resulting in decreased GnRH release and delayed or absent pubertal progression [5,53]. Epidemiological and cohort studies confirm that higher prepubertal fat mass is strongly associated with earlier puberty timing, while lower fat mass is linked to delayed onset [69,70]. In contrast, direct causal links between adiposity, neurotrophin signaling, and GnRH activation are primarily supported by animal models. Leptin acts as a permissive factor, but BDNF is required for the full activation of hypothalamic circuits governing reproductive maturation [31,53,71,72].

This dual pattern highlights neurotrophins as metabolic sensors capable of translating peripheral nutritional status into central reproductive signals. Through these mechanisms, neurotrophins help determine whether the body possesses adequate energy reserves to support the energetically demanding processes of pubertal growth and reproductive maturation.

## 5. Neurotrophins in Disorders of Pubertal Timing

### 5.1. Central Precocious Puberty

Neurotrophins—particularly BDNF—have been implicated in the pathophysiology of central precocious puberty (CPP), potentially contributing to premature maturation and activation of hypothalamic GnRH neurons [31,53,73]. BDNF expression increases in the arcuate nucleus during early puberty, and circulating BDNF levels are elevated in girls with CPP, suggesting that neurotrophin-driven hypothalamic activation contributes to early reawakening of the HPG axis [31,74]. Acting via TrkB receptors on GnRH and upstream KNDy neurons, BDNF enhances neuronal survival, differentiation, and excitability, directly stimulating GnRH expression, pulsatility, and secretion [7,31].

A growing body of evidence highlights a hypothalamic regulatory axis centered on the *FTO* gene, RNA methylation (m^6^A), and BDNF signaling [75,76]. FTO expression increases in the mediobasal hypothalamus during the peripubertal period and promotes m^6^A demethylation of *BDNF* mRNA, stabilizing transcripts and enhancing BDNF production. In turn, BDNF activates calcium-dependent and PI3K/Akt pathways, which upregulate GnRH expression and facilitate earlier puberty onset. Experimental overexpression of FTO or BDNF accelerates puberty in vivo, whereas knockdown delays its onset, supporting a mechanistic link between neurotrophin signaling and pubertal timing [31]. While the mechanistic framework of BDNF–FTO–GnRH interactions is largely supported by animal studies, emerging human data, including circulating BDNF measurements and genetic associations, provide translational support. Available human studies are generally limited by small sample sizes and cross-sectional designs.

These epitranscriptomic pathways intersect with metabolic and inflammatory cues. Childhood obesity and diet-induced hypothalamic inflammation activate microglia and astrocytes, disrupt leptin and insulin signaling, and converge on FTO/m^6^A and BDNF pathways. Within these inflamed circuits, BDNF contributes to synaptic remodeling and further increases GnRH neuron excitability [75,76]. Altogether, current evidence supports a role for BDNF as an integrator of metabolic, inflammatory, and neurotrophic signals associated with premature GnRH activation in CPP.

### 5.2. Delayed Puberty and Hypogonadotropic Hypogonadism

Neurotrophic signaling is essential for the development, migration, and function of GnRH neurons, and its disruption contributes to delayed puberty and congenital hypogonadotropic hypogonadism (CHH), including Kallmann syndrome. In contrast to CPP, evidence in delayed puberty and congenital hypogonadotropic hypogonadism includes both preclinical data and human genetic studies, particularly involving genes regulating GnRH neuron migration. During embryogenesis, neurotrophic factors guide GnRH neurons from the olfactory placode to the hypothalamus; defects in these pathways can impair this process and result in pubertal failure. Among the strongest genetic links, pathogenic variants in neuron-derived neurotrophic factor (NDNF) have been identified in patients with CHH and Kallmann syndrome. Animal models demonstrate that loss of NDNF disrupts GnRH neuron migration, leading to reduced GnRH availability and delayed or absent puberty [53,77,78]. Semaphorin signaling also plays a critical role in GnRH neuronal development. Genes such as *SEMA3A*, *SEMA6A*, and *SEMA7A* regulate olfactory axon guidance and the structural scaffolding required for GnRH neuron migration. Mutations in semaphorin ligands or their receptors (neuropilins and plexins) impair axonal pathways, alter vascular organization in the median eminence, and hinder hypothalamic innervation, producing phenotypes ranging from Kallmann syndrome to milder forms of delayed puberty [79,80,81,82]. Beyond NDNF and semaphorin pathway, the genetic architecture of delayed puberty and CHH appears largely oligogenic and remains incompletely defined.

At the opposite end of the spectrum, functional hypothalamic amenorrhea (FHA) represents a reversible form of GnRH deficiency in which environmental stressors—such as under-nutrition, psychological stress, or excessive exercise—suppress GnRH pulsatility. Individuals with FHA may carry heterozygous variants in CHH-related genes, supporting a model in which genetic predisposition interacts with environmental triggers to reduce GnRH drive. The Endocrine Society emphasizes that FHA remains a diagnosis of exclusion, yet genetic susceptibility appears increasingly relevant [83,84,85].

Genetic evaluation is recommended in adolescents with delayed puberty who present with anosmia, syndromic features, or a positive family history. Next-generation sequencing panels targeting neurodevelopmental and neuroendocrine pathways—including *NDNF* and semaphorin genes—are increasingly used in clinical assessment [53,86]. However, no neurotrophin-related biomarker is currently validated for diagnostic use. Research continues to explore whether neurotrophic pathways may eventually serve as therapeutic or predictive targets in disorders of pubertal timing [87,88,89]. A summary of neurotrophin-related mechanisms, implicated pathways, and clinical correlations across the major disorders of pubertal timing is provided in Table 1.

## 6. Therapeutic and Diagnostic Perspectives

### 6.1. Potential Therapeutic Modulation

The BDNF/TrkB pathway represents a compelling but still experimental target for therapeutic modulation of pubertal disorders, particularly those involving impaired GnRH neuron development or function. Although no approved therapies currently act directly on neurotrophin signaling, preclinical studies provide a mechanistic rationale for such an approach. During embryogenesis, GnRH neurons express TrkB, and BDNF directly promotes their neurite outgrowth, differentiation, and survival in vitro, supporting its neurotrophic role in GnRH system maturation [36]. In vivo, BDNF delivery increases hypothalamic GnRH and kisspeptin expression, elevates gonadotropin and sex steroid levels, and promotes oocyte maturation, indicating a conserved function across species [36,90].

Because native BDNF has poor pharmacokinetic properties and limited tissue specificity, therapeutic interest has shifted toward TrkB agonists. The small-molecule agonist 7,8-dihydroxyflavone has shown high-affinity binding to TrkB and activation of downstream signaling in neuronal models, with oral bioavailability and blood-brain barrier penetration in rodents. However, recent studies have questioned its selectivity, in vivo TrkB activation, and pharmacokinetic suitability, noting poor brain exposure and significant off-target effects, which limit its translational potential for hypothalamic or ovarian targeting [98,99,100].

TrkB-agonist antibodies such as Ab4B19 have demonstrated the ability to penetrate ovarian follicles after intravenous administration in mice, activate TrkB signaling, restore folliculogenesis, and normalize gonadal hormone levels in models of premature ovarian failure and aging. Ab4B19 was superior to BDNF in restoring oocyte number and quality, and it activated TrkB signaling in ex vivo human ovarian tissue. These antibodies exhibit tissue specificity for ovarian follicles and show promise for fertility restoration, but have not been tested in humans or for hypothalamic effects [101,102,103].

Adeno-associated virus (AAV)-mediated BDNF gene therapy offers the potential for tissue-specific, long-term BDNF expression. AAV vectors can be engineered for central nervous system or ovarian tropism, but clinical pharmacology is complex, with variable biodistribution, immune responses, and safety concerns such as genotoxicity and neurotoxicity. No studies have evaluated AAV-BDNF for pubertal or reproductive disorders in humans, and the approach remains experimental [104,105,106,107].

Collectively, these approaches—exogenous BDNF supplementation, small-molecule TrkB agonists, TrkB-targeting antibodies, and BDNF gene therapy—outline a potential therapeutic pipeline. Importantly, none of the currently proposed TrkB- or BDNF-directed strategies is close to clinical application for disorders of pubertal timing, and any intervention targeting neurotrophin signaling during brain and gonadal development carries significant theoretical risks that warrant extreme caution.

### 6.2. Neurotrophins as Biomarkers of Pubertal Disorders: Potential and Limitations

Peripheral BDNF shows developmental dynamics across childhood and adolescence and reflects, to some extent, the functional state of the HPG axis. In healthy populations, BDNF levels vary by pubertal stage, sex, age, body mass index, and—critically—platelet count, which is the main determinant of circulating concentrations [108,109]. In disorders of pubertal timing, BDNF tends to track extremes of GnRH activity: serum BDNF is elevated in CPP, whereas in FHA it is markedly reduced and exhibits a blunted circadian rhythm. Reduced BDNF has also been described in adolescents with major depressive disorder and adverse metabolic profiles, emphasizing the relevance of psychiatric and metabolic comorbidities [110,111].

These findings should be interpreted with caution given the substantial pre-analytical and biological variability of peripheral BDNF measurements. Despite these associations, BDNF is not yet a reliable clinical biomarker. Exercise markedly increases serum or plasma BDNF, with both acute and chronic training producing small-to-moderate elevations independent of menstrual cycle phase. Stress, metabolic status, and psychiatric conditions further confound interpretation. Pre-analytical variables (serum vs. plasma, clotting times, centrifugation, platelet activation) substantially alter measured levels, with serum capturing predominantly platelet-derived BDNF [108,109,112].

Pediatric studies remain limited by small sample sizes, cross-sectional design, and lack of validated diagnostic thresholds. Although BDNF tends to increase in CPP and decrease in FHA or severe psychiatric/metabolic states, these patterns are insufficient for clinical discrimination [110,111,112,113]. Genetic biomarkers are more promising: *NDNF* variants and related neurotrophic pathway genes are now included in CHH diagnostic panels [77,114].

Overall, neurotrophins currently function as mechanistic and research biomarkers, reflecting neurobiological states associated with GnRH activation or suppression, rather than as standalone diagnostic tools for pubertal disorders.

## 7. Challenges and Future Directions

Despite significant advances in understanding how neurotrophins regulate pubertal timing, several critical gaps remain. A limitation of the current literature is the predominance of preclinical evidence, with relatively few mechanistic studies available in humans. As highlighted by Jiménez-Puyer and colleagues, most work has focused on well-characterized hypothalamic populations—primarily GnRH and kisspeptin neurons—while the broader hypothalamic “ecosystem” in which these neurons operate remains poorly defined. The specific neuronal, glial, and metabolic circuits that integrate environmental, nutritional, and stress-related signals are still incompletely mapped [46]. Clarifying how neurotrophins shape these networks will require single-cell and spatial transcriptomics, advanced circuit-level imaging, and longitudinal human studies capable of linking cell-type–specific signatures to pubertal trajectories.

At the systems neuroscience level, adolescence has emerged as a sensitive period for experience-dependent plasticity. Laube et al. demonstrate that pubertal hormones exert region-, task-, and sex-specific effects on cortical plasticity, challenging the notion of a uniform relationship between endocrine changes and brain maturation [115]. Zanesco and Velloso’s review on BDNF and sex differences further underscores that BDNF signaling varies across brain regions, developmental phases, and biological sex, and is strongly modulated by lifestyle factors such as physical activity and stress [116]. Together, these findings highlight two key methodological priorities: (1) explicit sex stratification in studies of neurotrophins and puberty; (2) multimodal, longitudinal designs integrating endocrine measures, neurotrophins, neuroimaging, and behavioral assessments rather than relying solely on peripheral biomarkers.

From a genetic standpoint, the boundaries between classic pubertal disorders are becoming increasingly blurred. Self-limited delayed puberty and CHH share overlapping—though distinct—genetic architectures, with many patients harboring rare variants in genes involved in GnRH neuron development, migration, and function [117]. This suggests that neurotrophin-related genes may sometimes act as modifiers of susceptibility rather than straightforward monogenic causes. Hassani and colleagues summarize strong evidence implicating NDNF as an evolutionarily conserved neurotrophic factor essential for GnRH neuron migration, yet emphasize that current data remain fragmentary and require validation in both mechanistic and clinical settings [118].

Moving forward, several priorities will be essential:

- Functional characterization of neurotrophin-related variants, particularly NDNF and semaphorin pathway genes, using appropriate cellular and animal models.
- Longitudinal human cohort studies combining developmental endocrinology with neurotrophin profiling and brain imaging to track how neurotrophic pathways influence pubertal progression.
- Standardization of peripheral BDNF measurement protocols, encompassing blood processing, assay platforms, and normalization strategies—an essential step before any clinical application.

Only through these integrated approaches—spanning molecular biology, genetics, neuroscience, and clinical endocrinology—will neurotrophins be fully translated from promising mechanistic regulators into reliable, clinically actionable tools for understanding and managing disorders of pubertal timing.

### Limitations of Current Evidence

Current evidence linking neurotrophins to pubertal timing remains limited by several methodological constraints. Most mechanistic data derive from animal models, and human studies are frequently cross-sectional, underpowered, and focused on peripheral biomarkers that do not reliably reflect central neurotrophic activity. Measurement of circulating BDNF is particularly vulnerable to pre-analytical variability, platelet-derived contamination, and confounding by exercise, stress, psychiatric status, and metabolic health. Genetic evidence—although compelling for NDNF and selected guidance molecules—explains only a minority of congenital pubertal disorders and often lacks functional validation in appropriate cellular or in vivo systems [53]. Furthermore, the absence of standardized protocols for neurotrophin quantification and the scarcity of longitudinal pediatric cohorts limit the translation of neurotrophic findings into clinical practice [6,119]. Addressing these gaps will be essential to define the true diagnostic, prognostic, and therapeutic value of neurotrophins in pubertal disorders.

## 8. Conclusions

Neurotrophins, particularly BDNF and NDNF, emerge as central regulators of pubertal timing by linking early neurodevelopmental processes to hypothalamic network maturation and reproductive axis activation. Rather than acting as isolated growth factors, neurotrophins function as integrative signals that modulate GnRH neuronal plasticity and responsiveness to metabolic, hormonal, and environmental cues.

Across the spectrum of pubertal disorders, altered neurotrophic signaling contributes to distinct clinical phenotypes, from premature activation of the HPG axis in central precocious puberty to impaired GnRH neuron development in congenital hypogonadotropic hypogonadism and reversible suppression of GnRH pulsatility in functional hypothalamic amenorrhea.

Despite strong biological plausibility, neurotrophins currently remain research tools rather than clinical biomarkers or therapeutic targets, owing to limited specificity, confounding systemic influences, and incomplete human evidence. At present, their greatest clinical value lies in providing a conceptual framework to help clinicians interpret complex and heterogeneous pubertal phenotypes, rather than in guiding immediate diagnostic or therapeutic decisions. Future progress will rely on integrative and longitudinal studies combining neurobiology, endocrinology, genetics, and metabolism to define the translational potential of neurotrophin-based approaches in the diagnosis and management of pubertal disorders.

## Figures and Tables

**Table 1:** Summary of mechanistic and clinical evidence linking neurotrophins to specific pubertal disorders.

Pubertal Disorder	Neurotrophin(s) Implicated	Mechanistic Pathways	Data Evidence	Translational Relevance
Central precocious puberty [31,90]	BDNF	↑ Hypothalamic BDNF; TrkB–PI3K/Akt activation; FTO–m^6^A demethylation stabilizing BDNF; interaction with kisspeptin/KNDy circuits	Higher serum BDNF vs. controls; correlates with early GnRH pulsatility	Research biomarker; mechanistic insights but not clinically validated
Self-limited delayed puberty [53,91,92]	Mild neurotrophic involvement	Subthreshold variants in genes regulating GnRH neuron maturation	Partial overlap with CHH genes in some adolescents	Suggests modulatory rather than causal neurotrophic role
CHH/Kallmann syndrome [77,81,93]	NDNF, Semaphorin-related pathways	Impaired GnRH neuron migration; defective axon guidance; neurodevelopmental disruption	NDNF loss-of-function variants documented; anosmia + misrouted GnRH neurons	Strongest genetic link between neurotrophins and pubertal failure
Functional hypothalamic amenorrhea [94,95]	BDNF	Stress/energy deficit → reduced hypothalamic plasticity; estradiol–BDNF uncoupling	Markedly low circulating BDNF; blunted diurnal rhythm	Reflects reversible GnRH suppression; overlaps partially with CHH pathways
Metabolic pubertal disturbances (obesity/malnutrition) [54,96,97]	BDNF, NGF	BDNF–leptin interactions; diet-induced hypothalamic inflammation; altered excitatory/inhibitory balance	Associations between BDNF, adiposity, body mass index, and pubertal timing	Supports role of neurotrophins as metabolic sensors
Gonadal developmental alterations [55,62,90]	BDNF, NGF, NT-3	Regulation of folliculogenesis, spermatogenesis, steroidogenesis	Expression studies in human gonads; functional evidence in animal models	Highlights peripheral neurotrophin function beyond central nervous system

Abbreviations: BDNF, brain-derived neurotrophic factor; CHH, congenital hypogonadotropic hypogonadism; FTO, fat mass and obesity-associated; GnRH, gonadotropin-releasing hormone; KNDy, kisspeptin/neurokinin B/dynorphin; m^6^A, N6-methyladenosine; NDNF, neuron-derived neurotrophic factor; NGF, nerve growth factor; NT-3, neurotrophin-3; TrkB, tropomyosin receptor kinase B. ↑ indicates elevation.

## Data Availability

No new data were created or analyzed in this study. Data sharing is not applicable to this article.

## Abbreviations

The following abbreviations are used in this manuscript:

AAV	Adeno-associated virus
Akt	Protein kinase B
BDNF	Brain-derived neurotrophic factor
cAMP	Cyclic adenosine monophosphate
CHH	Congenital hypogonadotropic hypogonadism
CPP	Central precocious puberty
CREB	cAMP response element-binding protein
ERK	Extracellular signal–regulated kinase
FHA	Functional hypothalamic amenorrhea
FTO	Fat mass and obesity–associated
GABA	gamma aminobutyric acid
GnRH	Gonadotropin-releasing hormone
HPG	Hypothalamic–pituitary–gonadal
KNDy	Kisspeptin/Neurokinin B/Dynorphin
MAPK	Mitogen-activated protein kinase
m^6^A	N6-methyladenosine
NDNF	Neuron-derived neurotrophic factor
NGF	Nerve growth factor
NT-3	Neurotrophin-3
NT-4/5	Neurotrophin-4/5
p75NTR	p75 neurotrophin receptor
PI3K	Phosphoinositide 3-kinase
PLCγ	Phospholipase C gamma
STAR	Steroidogenic acute regulatory protein
TrkA/B/C	Tropomyosin receptor kinases A, B, and C
